# Kinesthetic and vestibular information modulate alpha activity during spatial navigation: a mobile EEG study

**DOI:** 10.3389/fnhum.2014.00071

**Published:** 2014-02-25

**Authors:** Benedikt V. Ehinger, Petra Fischer, Anna L. Gert, Lilli Kaufhold, Felix Weber, Gordon Pipa, Peter König

**Affiliations:** ^1^Neurobiopsychology, Institute of Cognitive Science, University of OsnabrückOsnabrück, Germany; ^2^Neuroinformatics, Institute of Cognitive Science, University of OsnabrückOsnabrück, Germany; ^3^Department of Neurophysiology and Pathophysiology, University Medical Center Hamburg-EppendorfHamburg, Germany

**Keywords:** spatial navigation, mobile EEG, alpha band, event related desynchronization, alpha suppression, virtual reality, independent component analysis, time-frequency analysis

## Abstract

In everyday life, spatial navigation involving locomotion provides congruent visual, vestibular, and kinesthetic information that need to be integrated. Yet, previous studies on human brain activity during navigation focus on stationary setups, neglecting vestibular and kinesthetic feedback. The aim of our work is to uncover the influence of those sensory modalities on cortical processing. We developed a fully immersive virtual reality setup combined with high-density mobile electroencephalography (EEG). Participants traversed one leg of a triangle, turned on the spot, continued along the second leg, and finally indicated the location of their starting position. Vestibular and kinesthetic information was provided either in combination, as isolated sources of information, or not at all within a 2 × 2 full factorial intra-subjects design. EEG data were processed by clustering independent components, and time-frequency spectrograms were calculated. In parietal, occipital, and temporal clusters, we detected alpha suppression during the turning movement, which is associated with a heightened demand of visuo-attentional processing and closely resembles results reported in previous stationary studies. This decrease is present in all conditions and therefore seems to generalize to more natural settings. Yet, in incongruent conditions, when different sensory modalities did not match, the decrease is significantly stronger. Additionally, in more anterior areas we found that providing only vestibular but no kinesthetic information results in alpha increase. These observations demonstrate that stationary experiments omit important aspects of sensory feedback. Therefore, it is important to develop more natural experimental settings in order to capture a more complete picture of neural correlates of spatial navigation.

## Introduction

Well-controlled studies under restricted laboratory conditions have contributed enormously to the knowledge about brain processes over the past decades. These insights are thought to capture relevant aspects of brain functionality that also hold true in natural settings or even generalize to brain processes under natural conditions. It remains to be tested whether these assumptions hold and to which degree the results obtained in reduced experimental setups transfer to natural conditions.

Specifically, such controlled settings often imply sitting in front of a computer monitor, thus omitting important sensory information that would otherwise be given in natural behavior. In particular in the case of spatial navigation, kinesthetic (registered by joint, tendon, and muscle proprioceptors) and vestibular sensory information (originating from translational or rotational changes mediated by the semicircular canals of the inner ear) have to be regarded as key percepts. With our present work, we attempt to set a first step toward evaluating the generalizability of typical laboratory paradigms to real world conditions.

In everyday life, navigation requires continuous multimodal integration of inputs from various senses—including visual kinesthetic, and vestibular information—to compute one's relative position in the environment. The mere ability to see already gives access to a multitude of spatial cues (e.g., optical flow, binocular disparity, or motion parallax) and aids not only to the recognition of objects but also to the perception of spatial relations. Vision, therefore, is assumed to dominate spatial processing. This can occur in various reference frames, i.e., multiple ways to describe how objects relate to each other. Several studies (e.g., Schicke et al., 2002, for review see Eimer, 2004; Pasqualotto and Proulx, 2012) provide evidence that even non-visual spatial perception via sound, touch, or proprioception is influenced by the existence of an early visually induced external reference frame when two modalities are interacting. Performance accuracies of healthy participants, as well as late-blind people, drop when additional biased sensory information of another modality poses a distraction. Congenitally blind people instead perfectly succeed in ignoring irrelevant stimuli exactly as the task requires. These results indicate that early visual experience establishes constitutive sensory integration within one common external reference frame and that this process does not emerge in the complete absence of vision.

However, some studies challenge the dominant role of vision. For example, Loomis et al. (1993) compared the path integration ability of congenitally blind and blind-folded sighted participants and found only small differences, suggesting that proficiency in spatial navigation relying on non-visual modalities is not necessarily dependent on previous visual experience. Other studies (e.g., Loomis et al., 1993; Klatzky et al., 1998; Wartenberg et al., 1998) also suggest that for accurate spatial updating, i.e., revision of internal information on the spatial context, vision alone is not sufficient when kinesthetic and vestibular signals that are normally generated by whole-body movements are missing.

Previous psychophysical experiments showed that when the availability of vestibular and kinesthetic sensory information was systematically varied, subjects' orientation estimates differ significantly: Frissen et al. (2011) found evidence of inaccurate spatial updating when kinesthetic information was provided but vestibular updating was prevented. Subjects tended to underestimate their perceived self-motion while they were walking in place on a circular treadmill in the absence of vision. The authors hypothesized that this effect potentially results from the conflicting zero-movement input from the vestibular system. In contrast to this, passive movement generated by the treadmill provided only vestibular information but yielded accurate spatial updating in spite of the complete absence of muscle activity. While vision was absent in the study of Frissen et al., Chance et al. (1998) showed that performance in indicating location directions of previously passed objects benefited when vestibular and kinesthetic information were provided in addition to vision (Chance et al., 1998). This is not surprising if one regards the following: Usually, proprioceptive information does not necessarily have to be available or congruent when vestibular information is changing, for example when driving a car, riding a train, or being carried as an infant. Muscle activities during natural movements instead never occur without appropriate vestibular updating. Likewise, many other psychophysical experiments (Chance et al., 1998; Loomis et al., 1999; Kearns et al., 2002; Frissen et al., 2011) show that altering the availability of sensory information causes changes in behavior. Therefore, we also expect to see modulations of the underlying brain processes.

A number of studies investigated the electrophysiological correlates of spatial navigation by recording electroencephalography (EEG) (e.g., Gramann et al., 2006, 2010; Plank et al., 2010; Chiu et al., 2012). Gramann et al. (2010) distinguished between neuronal correlates of subject groups that either use allocentric or egocentric reference frames while navigating. Subjects were classified according to their strategies of mentally representing their heading in a given environment (Gramann et al., 2006, 2010; Goeke et al., 2013). Usually, roughly half of all subjects hold on to an egocentric reference frame, which is also named *Turner* strategy, while the other half solves the navigation task according to a *Non-Turner* strategy using an allocentric reference frame. This nomenclature comes from the fact that so-called *Turners* update their heading and position in a mental map, whereas *Non-Turners* only update their position but not their heading direction—they will “not turn away” from their initial orientation. This updating is also influenced by response modality (Avraamides et al., 2004) and it depends on whether translational or rotational aspects are included (May, 2004), and whether subjects actively move through the environment (Klatzky et al., 1998). In addition to the predicted behavioral differences between these two groups, Gramann et al. (2010) also detected significant differences in their neuronal activities. Subjects virtually passed through a tunnel consisting of a straight segment, a turn of varying angle, and another straight segment while EEG was measured. During turns, alpha desynchronization occurred in parietal and occipital areas, which is in general considered to reflect enhanced cognitive processing in the respective areas (e.g., Pfurtscheller and da Silva, 1999). Gramann et al. (2010) moreover reported stronger alpha blocking in Turners in right inferior occipital gyrus, whereas Non-turners showed a stronger alpha blocking near bilateral occipito-temporal, inferior parietal, and retrosplenial cortex. The authors argue that this enhanced suppression probably indicates abstract processing of egocentric visual flow (like using a bird's eye view) when maintaining an allocentric reference frame. Functional magnetic resonance imaging studies provide similar evidence for activity in parietal cortex, more precisely in the precuneus and retrosplenial cortex (Committeri et al., 2004; Wolbers et al., 2007).

The studies described above, however, are conducted in stationary setups and therefore have provided insights only into neural processing of spatial updating without physical movement. For this reason, the authors emphasize the need for whole body imaging under real world conditions (Gramann et al., 2014). Following these ideas, we test in what way these findings on brain processes during passive navigation generalize to a task that provides not only visual input, but successively adds proprioceptive and vestibular information—two major additional task-relevant senses.

The investigation of physiological mechanisms of spatial navigation raises challenging technical issues that go hand in hand with the application of non-invasive techniques to study the human brain while the subject is in motion. Techniques such as functional magnetic resonance imaging or positron emission tomography have been used extensively in spatial navigation research (e.g., Maguire et al., 1998; Committeri et al., 2004; Wolbers et al., 2011) and provide a high spatial resolution, but are unsuitable for mobile setups as they are stationary. Miniaturization of electronic devices has led to the development of mobile EEG recording equipment; yet, EEG signals are weak and prone to movement artifacts. Recently developed systems equipped with actively shielded electrodes and cables have been particularly designed to record electrophysiological data from moving and even walking probands (Waveguard, ANT, Netherlands). Furthermore, advances in data analysis techniques permit improved cleaning of EEG signals from artifacts, instead of excluding such recordings (Gwin et al., 2010; Delorme et al., 2011). The aim of our study is to take advantage of these new possibilities to extend previous findings on neural correlates of spatial navigation and to investigate especially the integration of multiple senses, which inevitably occurs during navigation coupled with active movement.

We complemented the EEG system with a mobile virtual reality device that allowed us to implement a less restricted but at the same time well-controlled experimental paradigm. The core of our experiment is to vary the availability of vestibular and kinesthetic sensory information while the provided visual input stays identical across conditions.

To this end, we devised a specific hardware built of two straight segments that are connected by a turnable platform. The segments can be rotated freely to form new path configurations and serve as guide rails to keep our subjects on track. A cart assured stability and carried auxiliary technical equipment. Additionally, it enabled us to transport subjects passively along the track preventing kinesthetic updating in the presence of vestibular information. Our main motivation for devising the construction was to get access to the manipulation of vestibular updating with the help of the turntable: It can be rotated by leg movements of the subject while the orientation of the upper body remains constant providing kinesthetic input but no vestibular updating (see Materials and Methods for a detailed description).

Considering that our task implied similar sensory modifications, we were interested in a comparison between our subjects' behavior and the studies introduced earlier (Chance et al., 1998; Frissen et al., 2011): Providing vestibular information leads to better performance, therefore, we hypothesized that the performance of our subjects, namely the accuracy of their homing angle estimates, would improve with vestibular sensory information. Furthermore, in the passive baseline condition, we also expected to detect alpha suppression in the same regions as Gramann et al. (2010). Adding only kinesthetic or vestibular information could lead to an incongruency effect and consequently higher alpha suppression, reflecting increased cognitive demands. Since subjects are probably more involved and “immersed” in the active conditions with additional sensory input, we assumed to find even stronger effects there. This hypothesis is also suggested by previous comparisons of EEG recordings of participants in 3D or 2D environments, which showed a very similar alpha decrease in the more immersed 3D setting (Havranek et al., 2012; Kober et al., 2012).

Taken together, the overall aim of our study was to assess how previous findings on EEG correlates of spatial navigation extend to life-like experimental tasks and, moreover, to explore how the integration of multiple senses influences the underlying task-driven brain dynamics.

## Materials and methods

### General methods

#### Subjects

Five right-handed male students (mean age: 22.4 years, range 21–24 years) participated in the study. Two of those subjects showed allocentric navigation behavior in a previous online test (www.navigationexperiments.com/TurningStudy.html), while the other three exhibited egocentric behavior. Subjects' gaming experience has been either less than 6 months (S3, S4), 2–5 years (S5), or up to 10 years (S1, S2). All participants had normal or corrected to normal vision. They were paid 8€ per hour. The procedure had been approved by the local ethics committee, and prior to the start of the experiment subjects gave informed written consent.

### Design

We employed a 2 × 2 within-subjects design by manipulating available information as follows: (1) In the passive condition, participants stood while watching a movement presented via a head-mounted display. (2) In the vestibular condition, subjects were moved while standing on a cart and thus received vestibular but no kinesthetic information about the turn. (3) In the kinesthetic condition, subjects were rotating a turntable beneath their feet with a lower limb movement while keeping their head oriented straight. This mimics an on-the-spot-turn without vestibular updating but appropriate kinesthetic information. (4) Lastly, the active condition approximated natural behavior best as participants walked and turned by themselves.

#### Task

Our experiment is based on a modified triangle completion task: Participants traversed one leg of a triangle, turned on the spot, and continued along the second leg. In order to keep our probands on the right track when navigating through a random-dot starfield they had to follow a centered, small, spherical guiding object from their start to their end position. The starfield consisted of randomly distributed dots that were aligned on a horizontal ground plane. Additionally, a small number of dots were scattered across the remaining space, leaving the area through which the subjects were passing clear (see Supplementary Materials). Visibility of the dots faded to black within 20 m viewing distance from the subjects. At the end of each trial, a virtual arrow faded in with a black background while the starfield dissolved. The arrow was displayed at a constant distance in front of the participants. It was initially oriented in walking direction, and subjects rotated it either to the left or to the right by pressing two buttons on a gamepad. Once the arrow reached its desired direction, the participants pressed a third button to confirm their final decision.

Each subject completed 120 trials per condition. A total number of 480 trials per subject were recorded in four sessions with two 60 trials blocks and therefore two different conditions in each session. Participants were allowed to take breaks in between the blocks or whenever requested. Each of the six different angles (30°, 60°, 90°, to the left and to the right) occurred equally often in each condition, namely 20 times. The order of the conditions across subjects as well as the order of the angles in the conditions was randomized. All subjects took part in a training session prior to their first recording in which they familiarized themselves with the setup and task in each condition until they felt confident with the experiment.

#### Hardware

**Figure 1:**
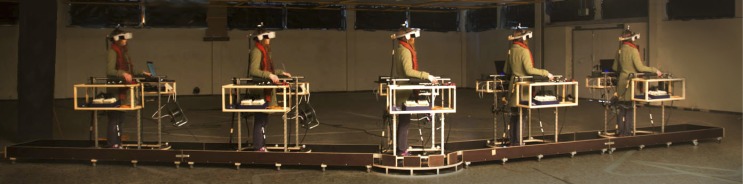
**Walkway.** We devised a flexible walkway consisting of two straight segments that are connected by a turnable platform. Subjects were wearing an EEG cap (not shown in the picture) and a Head Mounted Display, and (were) moved along this predefined track while being stabilized by a cart, carrying auxiliary equipment.

**Figure 2:**
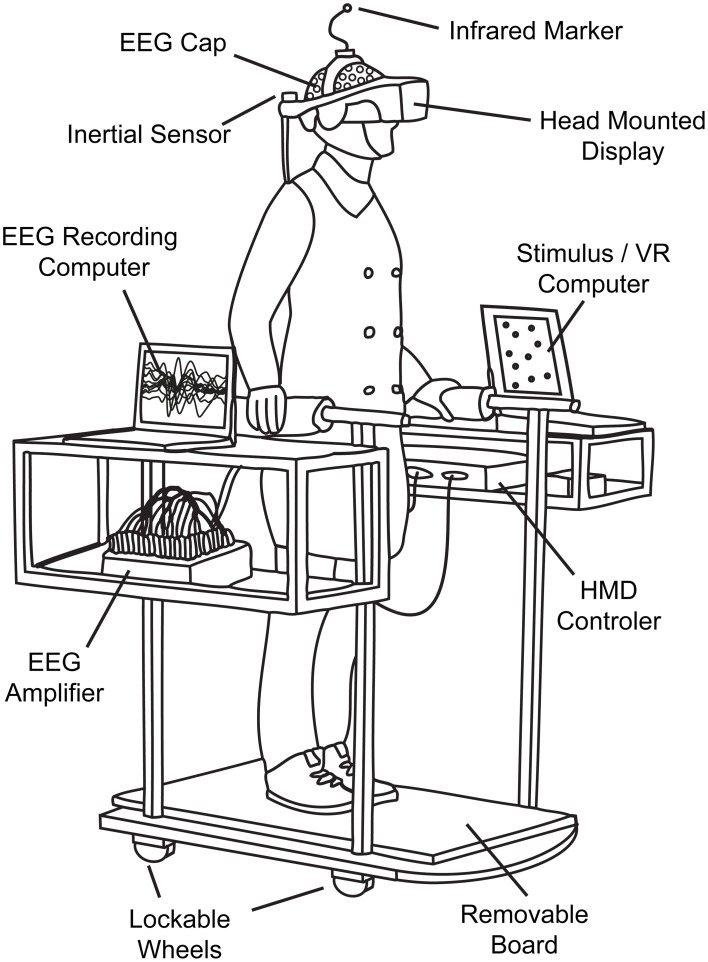
**Technical setup.** The cart ensured that subjects stayed on track and completed the predefined path as required. It furthermore served as convenient repository for auxiliary mobile VR and EEG equipment like laptops or amplifiers.

In each of our four conditions, subjects were moving or standing on a customizable walkway consisting of two straight segments which are linked by a round platform with a turntable that can be locked in position (Figure 1). The straight segments have wheels attached and can be adjusted by the experimenter between trials to match the new path layout and serve as guide rails. Participants moved along the track inside of a wheeled walking frame in order to prevent deviations from the desired path. The cart additionally provided storage space for VR and EEG equipment (Figure 2).

The virtual environment was developed in Python-based WoldViz Vizard and conveyed via a Head Mounted Display (HMD, nVisor SX60, total horizontal field of view = 44°). Positions were tracked with the optical PPTX4 Precision Position Tracker system (WoldViz). We used two trackers—one was attached on top of the HMD to assess the subject's position and the other one was placed at the end of the second straight segment to determine the exact angle between the two segments prior to the beginning of each trial. The angle was calculated in relation to the center of the virtual environment that had been previously set to the center of the turntable. Precise knowledge about the accurate track and the required angle of the turn was essential as this information was displayed to the subject via the guiding object in the virtual environment. The subjects' head orientation was tracked with an additional inertial orientation sensor (InterSense InertiaCube2+) that was directly attached to the HMD. In order to transfer the rotation information of the turntable to the displayed virtual environment—which was required for the kinesthetic condition—a second wireless inertial 3D motion tracker (Xsens MTw) was attached underneath the platform.

For adjusting the arrow at the end of each trial, subjects used a consumer gamepad (Microsoft Sidewinder Plug and Play Gamepad). All devices were connected to a laptop (Dell Precision M4700, i7-3720 2.6 GHz, 4 GB Ram, NVIDIA Quadro K2000M) that rendered the virtual environment, transmitted it to the HMD via a video control unit (NVIS) and simultaneously sent triggers to the EEG laptop (DELL Latitude E6230, i5-3320 2.6 GHz, 4 GB Ram).

### Statistical analysis of behavioral data

**Figure 3:**
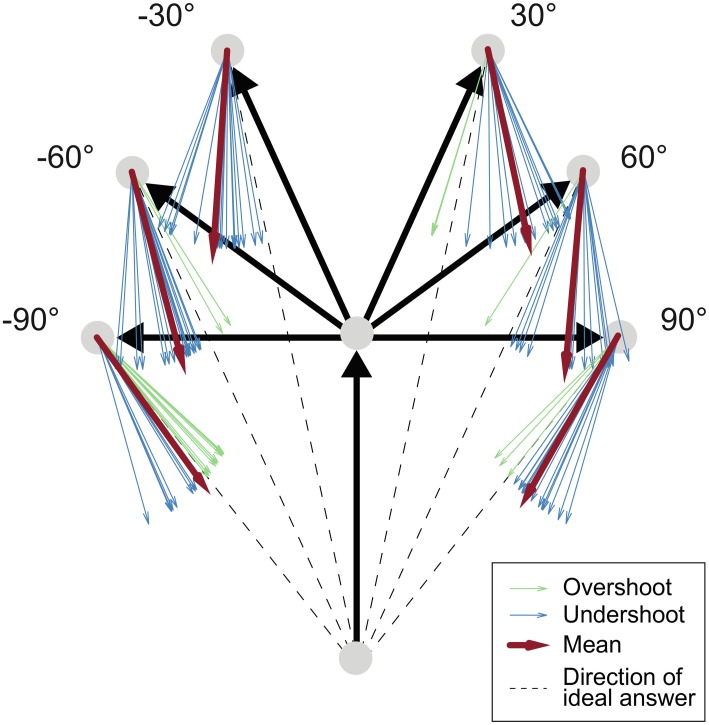
**Task schema and exemplary data.** Black arrows: Traversed path. Dashed line: Ideal response. Blue arrows: Underestimated answers (exemplary answers from one single subject in the active condition). Green arrows: Overestimated answers. Red arrows: Mean answer angles. Comparing the difference between the red mean arrow and the dashed line in the 30° and 90° turning angle configurations shows that the amount of underestimation was less pronounced with increasing turning angle.

By using the law of sines, the correct homing angle—defined as the direct line between start and end position (see Figure 3)—was calculated from the exact angle of the on-the-spot turn and the three vertices of the triangle: the start position, the position of the turn and the subjects' end position where the answer was given. The correct answer was then subtracted from the estimated angle, yielding negative errors for responses that underestimated the correct angle. Such underestimation, also called *undershoot*, is indicated via an arrow that was not rotated far enough under the assumption that the shortest way was used. In Figure 3 all blue arrows display underestimation behavior. Correspondingly, positive errors that are shown in green denote an arrow adjustment that ended beyond the correct angle under the assumption of the shortest route, which corresponds to so-called *overestimation behavior*. Overestimation of the correct answer angle, or overshoot, hence corresponds to an inward bias in a triangle-completion task. We will call this measure *relative error* which equates the systematic bias of a subject by including negative and positive signs, and distinguish it from *absolute errors*. Absolute error simply refers to the absolute amount of the error irrespective of any knowledge about over- or underestimation of the error.

In a next step the data were winsorized, setting all values beyond the 5th or 95th percentile to the nearest percentile in order to get robust estimates of the mean errors. This was done separately for each subject-condition combination to avoid raising the performance of a single subject or a single condition. Two 2 × 2 repeated measures analyses of variance (ANOVA) with mean relative error and variance of relative errors across trials as dependent variable were conducted in SPSS (IBM) with two factors: kinesthetic information (on/off) and vestibular information (on/off). As the most extreme winsorized relative errors were −69.9° and 41.4°, implying that the von Mises distribution can be approximated by a normal distribution (Mardia and Peter, 2000, p. 36), circular statistics were not required.

Changes of variance across conditions were investigated by computing multiple-sample and pairwise Levene's tests on the unwinsorized data of all five subjects individually. This assessment is of special interest to us as a difference in variance could be regarded as change in stochastic error. Furthermore, we correlated the absolute errors with the trial numbers to check for general performance improvements. To detect a potential change in the over-/underestimating behavior we additionally correlated the relative errors with the trial numbers.

### Physiological methods

#### Recording and preprocessing

Electrophysiological data were recorded using 128 Ag/AgCl electrodes which were placed according to the 5% international system (Oostenveld and Praamstra, 2001). We kept scalp impedances below 10 kOhm and sampled EEG data with 1024 Hz using an average reference (asalab, ANT, Netherlands) with the ground electrode placed on the forehead. The electrode positions were digitized using a 3D positioning device (Xensor, ANT, Netherlands). We used passive electrodes that are actively shielded (Waveguard, ANT, Netherlands), which minimizes cable sway and line noise artifacts.

We analyzed the EEG data with custom scripts using MATLAB (Mathworks) and EEGLAB (v12, SCCN, Delorme and Makeig, 2004). The data were resampled to 256 Hz and filtered with a 1 Hz high-pass (−6 dB cutoff: 0.5 Hz, 1 Hz transition bandwith) and a 120 Hz low-pass (−6 dB cutoff: 124 Hz, 8 Hz transition bandwith) FIR filter (EEGLAB, firfilt plugin from Widmann). In order to counter line noise, a notch FIR filter between 48 and 52 Hz (−6 dB cutoff: 49 Hz, 51 Hz, 2 Hz transition bandwidth) was applied. The data were visually cleaned for strong artifacts resulting from electrical noise and strong muscle artifacts. On average 9.5% of trials (range=[0% 26.7%]) were excluded from the analysis. Moreover, channels with extreme noise or signal drop-off were removed. On average 4.25 channels (range = [2 13]) were excluded. The data were re-referenced to the new average of all remaining data channels. As re-referencing to the average introduces correlations to the data, channel IZ was excluded in all subjects to get a rank complete data matrix. The AMICA algorithm (version 12, Palmer et al., 2008) was applied with standard parameters except from the addition of automatic rejection of unlikely data. In total we obtained 5485 clusters.

Dipoles of each IC-topography were fitted using the DIPFIT toolbox (Oostenveld and Oostendorp, 2002) and a standard Boundary Element Method (BEM). Individual electrode positions were warped to fit to the template. When the explained dipole variance was less than 85%, or the source localization was outside of the brain, indicating neck muscle or eye artifacts, ICs were excluded from the analyses. In total, the remaining set of ICs consisted of 1807 components.

#### ERSPs and clustering

After epoching the data from −20 s before the turn to 12 s after the turn, event related spectral perturbations (ERSPs) were calculated using three-cycle Morlet wavelets on the lowest frequency linearly increasing to 75 cycles at 50 Hz. We accounted for different trial lengths by linearly warping the ERSPs in the time domain (see Gwin et al., 2010). The duration of the central part of the first straight leg and the complete turn was warped to a constant time span of 2.5 and 4.25 s, respectively. After warping, we applied single trial normalization (Grandchamp and Delorme, 2011), i.e., we divided each point in time during the turn segment by the mean log power of the baseline segment of that specific trial, which is the central part of the first straight leg. Finally, trial average ERSPs were calculated to serve as input for subsequent clustering.

ICs were grouped into functional and anatomical clusters to allow a comparison of components over subjects and sessions. After grouping, principal component analysis (PCA) was applied to reduce measures (ERSP, scalp maps, and dipole location) into a joint measure space. We used the standard EEGLAB k-means clustering to obtain functional clusters of ICs over subjects. IC-clustering parameters from previous studies (Gramann et al., 2010) were used: 3D dipole locations were weighted by a factor of 15, ERSPs were reduced by PCA to 10 dimensions, normalized and weighted by a factor of 4. As an additional measure, IC topographies were reduced to 10 dimensions, normalized and weighted with 1. Finally, a PCA dimension reduction to 10 dimensions was applied to the joint measure space. This combined joint measure space over all subjects was clustered with a robust k-means algorithm into 25 clusters plus an additional one that contained outliers deviating by more than 3 standard deviations.

#### Cluster-stability test

In order to test the robustness or stability of our clusters, we compared them against the H_0-hypothesis that they are not stable; or in other terms, as k-means clustering always returns k clusters, we have to make sure that each cluster is not a random result. Thousand bootstrap samples with 1807 ICs in each sample were drawn with replacement from the set of all ICs. The same clustering procedure (as described above) was used in order to get a bootstrap distribution of clusters. We then calculated the maximal overlap of the bootstrapped cluster components with the originally observed cluster components. The overlap was calculated as the number of identical components in both clusters. We also calculated a normalized overlap where we removed multiple identical components in the bootstrap clusters. This did not change the results. Afterwards, we calculated the H_0_-distribution by assuming that the clusters were randomly arranged in the brain: The same bootstrapping procedure was used, but we randomly applied cluster labels to the ICs, assigning them to random clusters. In a last step we tested both distributions of overlap values against each other with an unpaired *t*-test. All clusters were significantly different from their H_0_-distribution (*p* < 0.001). As we still found the same clusters after resampling, we showed that our clusters were stable and not randomly assigned.

#### ROI analysis

As described above, we were interested in cortical alpha band modulation during the turn, which has been shown to be sensitive to spatial updating (Gramann et al., 2010). We defined a region of interest (ROI) defined in time-frequency space consisting of the turn from 0 to 4250 ms and the alpha band with its well-established borders of 8 and 12 Hz. Then we analyzed the clusters based on their component ERSP ROI activations. To check whether the activations in the ROI differ significantly from zero, we Monte Carlo resampled data points with replacement 1000 times and calculated their means. Finally, we calculated the *p*-value by dividing the number of values that are larger (respectively smaller) or equal to the observed mean by the total number of values. To compensate for two-sided testing, resulting values were multiplied by two to get the respective *p*-values.

To test for differences between conditions, we used the EEGLAB “statcond” function (Delorme and Makeig, 2004) and applied a non-parametric permutation-based 2 × 2 ANOVA to our data. For *post-hoc* investigations of differences between conditions we used permutation-based unpaired *t*-tests.

#### Cluster selection

To select those clusters that are informative for our hypothesis, we deployed the following strategy: First, artifactual clusters representing muscular, oculomotor, or cardiac activities were identified by their dipole locations and spectra. We excluded these clusters from further analyses. The remaining clusters were screened for modulations in the alpha band during spatial updating that were similar to those previously reported (Gramann et al., 2010). Further details on cluster selection will be given in the results section.

## Results

### Behavioral results

In the following section, we will compare subjects' pointing errors across the four different conditions when performing a modified triangle-completion task.

The average trial duration including the time of the response was 36.4 s. Traversing the straight segments required on average 7.8 s for the first and 7.6 s for the second segment. The mean duration of all turns was 5.0 s and the average response time for rotating the arrow was 7.5 s (passive condition: 6.2 s, kinesthetic condition: 8.3 s, vestibular condition: 7.9 s, active condition: 7.7 s). Pairwise *t*-tests result in significant differences between the passive and all other conditions (against kinesthetic: *p* = 0.003, against vestibular: *p* = 0.015, against active: *p* = 0.010). However, the reduced response times in the passive condition are not surprising, as the subjects did not have to stop the cart (kinesthetic/active condition) or wait for the cart to be stopped (vestibular condition) in order to answer.

After classifying all trials into either Turner or Non-Turner responses, we found that 98.8% of all given answers were closer to the optimal Turner response. It seems reasonable to assume that the remaining 1.2% of all trials were merely highly erroneous trials than genuine Non-Turner responses. Therefore, we conclude that regardless of their previously determined preference, our subjects responded in an egocentric reference frame in the present study.

The averages of the absolute errors over trials were 13.3°, 14.3°, 12.8°, and 12.2° in the passive, kinesthetic, vestibular, and active condition, respectively. Whether the absolute errors differed significantly between conditions was assessed by a 1 × 4 and a 2 × 2 repeated measures ANOVA. None of the two tests showed any significant effects.

Means over subject averages and bootstrapped 95% confidence intervals of relative errors for the four conditions were −8.6° [−21.2°, −3.5°], −1.1° [−13.8°, 6.0°], −6.3° [−11.8°, −0.7°], and −7.4° [−13.3°, −1.7°]. The negative sign indicates a tendency toward undershooting the correct answer angle in all but the kinesthetic condition.

In Table 1, means of the systematic relative error and standard deviation of all trials are shown for each subject in each condition. It denotes rather heterogeneous behavior between subjects concerning angle estimation accuracy and performance changes across conditions.

**Table 1:** **Mean errors and standard deviations [mean (std)] for each participant in every condition**.

	**Passive**	**Kinesthetic**	**Vestibular**	**Active**
S1	−3.9 (10.4)	−2.6 (19.2)	−13.7 (15.5)	−15.8 (17.3)
S2	−7.7 (17.5)	9.9 (21.2)	−6.6 (28.3)	−3.2 (15.9)
S3	−1.6 (6.4)	5.6 (8.6)	1.4 (6.2)	−0.7 (17.8)
S4	−25.5 (27.5)	−21.3 (28.8)	−12.6 (26.1)	−15.8 (20.3)
S5	−4.1 (21.1)	2.7 (17.0)	0.3 (11.7)	−1.6 (9.9)

Correlations of the absolute errors with the trial numbers showed an improvement in performance over the whole experimental course in four of five subjects (S1: *r* = −0.387, *p* < 0.001; S2: *r* = −0.308, *p* < 0.001; S4: *r* = −0.326, *p* < 0.001; S5: *r* = −0.139, *p* = 0.003; S3, ns: *r* = −0.068, *p* = 0.150).

The undershooting behavior of four of the five subjects was similarly reduced over time according to the correlations between relative errors and trial numbers (S1: *r* = 0.464, *p* < 0.001; S3: *r* = 0.385, *p* < 0.001; S4: *r* = 0.359, *p* < 0.001; S5: *r* = 0.483, *p* < 0.001; S2, ns: *r* = 0.068, *p* = 0.189). Thus, subjects showed small learning effects after prior training.

A 2 × 2 repeated measures ANOVA with kinesthetic sensory information (on/off) and vestibular sensory information (on/off) as factors and the mean relative errors over all 120 trials for each subject and condition as dependent variable revealed a significant interaction [*F*_(1, 4)_ = 24.42, *p* = 0.008, partial η^2^ = 0.859] but no significant main effects [kinesthetic: *F*_(1, 4)_ = 2.661, *p* = 0.178, partial η^2^ = 0.243; and vestibular: *F*_(1, 4)_ = 0.337, *p* = 0.593, partial η ^2^ = 0.078]. In each factor level, the dependent variable was normally distributed.

**Figure 4:**
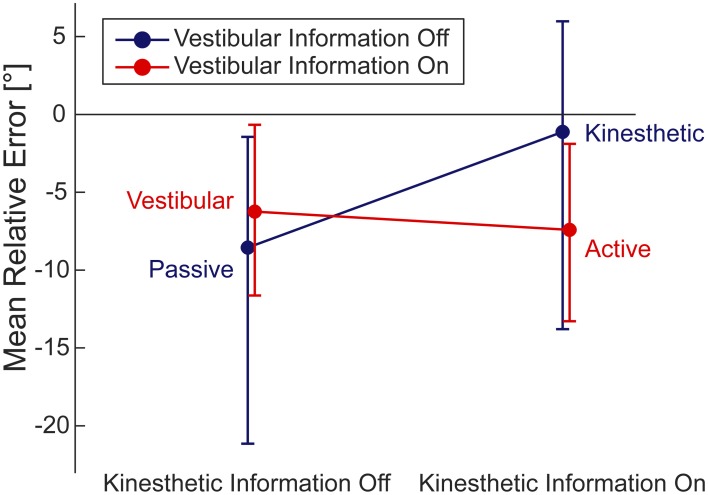
**Interaction of the mean relative errors in the respective four conditions.** Within 2 × 2 ANOVA (95% CI). By inspecting the 95% CIs we find underestimation of the correct homing angle (i.e., negative relative error) in all conditions but the kinesthetic one.

The interaction plot (Figure 4) shows that the subtraction of vestibular information from active walking resulted in a much less pronounced or nearly absent bias toward underestimating the correct homing angle in the kinesthetic condition. In contrast to this, the subtraction of vestibular information from the vestibular to the passive condition did not evoke such a drastic change. The kinesthetic condition, therefore, seems to be special in regard to the systematic performance error of our subjects. However, multiple comparison corrected pairwise *t*-tests between individual conditions yielded no significant differences.

With subject-specific variances of relative errors across trials as dependent variable, the 2 × 2 repeated measures ANOVA yielded no significant effects. In order to examine how random errors of individual subjects differed across conditions, we calculated robust multiple-sample Levene's tests for equal variances for each subject. The tests indicated that only for three subjects variances were heterogeneous in at least two conditions. After conducting pairwise Levene tests for each subject, we found diverging multiple comparison corrected significant differences (α = 0.0083) for the different subjects. The effects were diverging as they were either only detected in single subjects (for passive-kinesthetic, passive-vestibular, and kinesthetic-vestibular condition comparisons) or otherwise were present in two participants but contradicting each other as the differences of the respective two variances had opposite signs. Subject 4 exhibited a higher variance and thereby stochastic error in the passive condition compared with the active condition, whereas for subject 1 it was exactly the other way round. These results let us conclude that a change in condition does not lead to a systematic change in stochastic error in our group of five subjects.

### EEG results

During the experiment, subjects needed to update their spatial heading and position to point back to their starting position. We expected to detect the strongest effects of spatial updating processes during the turn. In order to investigate alpha-band related modulation during the turn we calculated time frequency (ERSPs) decompositions of our EEG data.

The EEG data were clustered into 25 individual clusters, not only to remove artifactual components, but also to identify separate electrophysiological processes. Due to the low number of participants we can only claim statistical evidence for our group and not the whole population.

We visually inspected the cluster spectra and dipole locations for the purpose of locating non-neural artifact clusters and identified six stereotypical muscle clusters, one heart and one eye artifact cluster. We also detected one theta-midline cluster (Onton et al., 2005). Seven further clusters did not show any sign of specific alpha modulation in the ROI and we could not classify them as other electrophysiological processes (see Table 1, Supplementary Material). We excluded these and the previously mentioned artifact clusters from further analyses.

**Figure 5:**
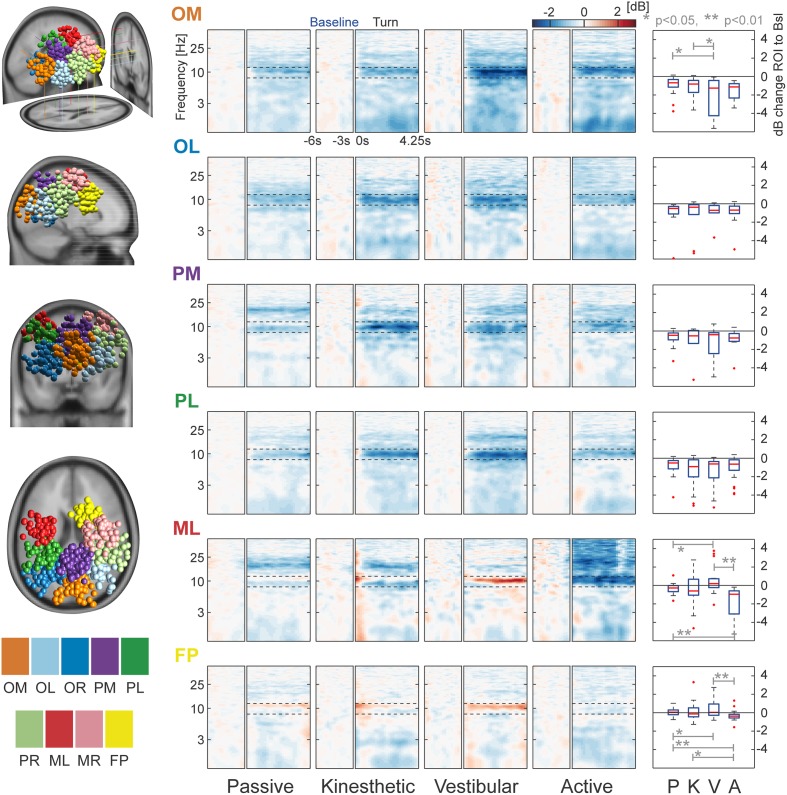
**Clusters and task-related alpha modulations.** The left column displays the locations of all dipoles belonging to the nine separate clusters projected to an MNI standard brain. Log-frequency ERSPs are shown in the central four columns. Blue denotes a decrease and red an increase in EEG power compared with baseline. ERSPs are not shown for PR, MR, and OR—they strongly resemble the pattern of their contralateral equivalents PL, ML, and OL. Boxplots in the right column depict the mean, cluster-wise ERSP activity in the alpha band (8–12 Hz) during the turn for each condition. In cluster OM the vestibular condition differs significantly from that in the passive and kinesthetic condition. More anterior clusters ML and FP show significant alpha band effects between vestibular and passive, vestibular and active, and active and passive conditions. ERSP alpha activity of cluster FP shows a significant difference between the kinesthetic and active condition. The clusters are labeled as follows: OM, Occipital Medial; OL, Occipital Left; OR, Occipital Right; PL, Parietal Left; PM, Parietal Medial; PR, Parietal Right; ML, Motor Left; MR, Motor Right; and FP, Fronto-Parietal.

The remaining nine clusters [Occipital Medial (OM), Occipital Left (OL), Occipital Right (OR), Parietal Left (PL), Parietal Medial (PM), Parietal Right (PR), Motor Left (ML), Motor Right (MR) and Fronto-Parietal (FP)] were analyzed in more detail. Table 2 shows the coordinates of the cluster centroids and their localization in the brain; they are, due to the large spread, not necessarily representative for the exact location of the underlying source (Akalin Acar and Makeig, 2013). All nine clusters can be seen in the left section of Figure 5. The ERSPs of PR, MR, and OR are not shown as they exhibit similar (PR) to identical (OR, MR) patterns over conditions to their contralateral equivalent.

**Table 2:** ***X, Y, Z* coordinates in Talairach space (Lancaster et al., 2000) of the cluster centroids and their localization in the brain**.

**Cluster name**	***X, Y, Z* Talairach coordinates**	**Anatomical structure**	**Broadmann area**
OM	5.28, −81.79, 25.73	Cuneus	Broadmann area 18
OL	−30.52, −62.62, 14.96	Middle temporal gyrus	Broadmann area 10
OR	32.39, −61.21, 18.21	Middle temporal gyrus	Broadmann area 39
PL	−34.40, −39.73, 43.91	Inferior parietal lobule	Broadmann area 40
PM	3.08, 50.80, 43.97	Precuneus	Broadmann area 7
PR	41.98, −33.81, 34.17	Inferior parietal lobule	Broadmann area 40
ML	−38.37, −8.14, 52.29	Precentral gyrus	Broadmann area 6
MR	34.61, −12.58, 58.40	Precentral gyrus	Broadmann area 6
FP	20.65, 6.99, 32.77	Cingulate gyrus	Broadmann area 32

As a first step of analysing the selected clusters, we looked at individual cluster data pooled over all conditions in order to investigate alpha modulation in the ROI. We observed a significant alpha decrease in all occipital/parietal clusters as the bootstrapped means were significantly different from zero (*p* < 0.001). This replicates the findings of previous studies (Gramann et al., 2010; Plank et al., 2010; Chiu et al., 2012). Remarkably, we do not find significant alpha decrease in the other, more anterior clusters (MR: *p* = 0.518, ML: *p* = 0.092, and FP: *p* = 0.306).

A main question of the study was the investigation of alpha modulation between different conditions. We therefore pooled over all clusters that individually showed a significant alpha decrease (OM, OL, OR, PM, PL, PR). A significant effect of the factor vestibular (*p* = 0.017) and a significant interaction (*p* = 0.006) was found using a bootstrapped 2 × 2 ANOVA. *Post-hoc* comparisons with Monte Carlo permutation unpaired *t*-tests showed a significant difference of the passive against the kinesthetic condition (*p* = 0.039), the passive against the vestibular condition (*p* = 0.001), and the active against the vestibular condition (*p* = 0.017). These results indicate that the passive condition does not generalize to all other conditions, as the kinesthetic and the vestibular conditions go along with alpha suppression that is stronger than in the passive condition.

In order to check whether we find differences in single clusters between at least two conditions, we split the data into clusters and conditions and applied a permutation-based ANOVA. The factor kinesthetic and the interactions were significant in two of the three anterior clusters [ML(Kinesthetic): *p* < 0.001, ML(Interaction): *p* = 0.011, FP(Kinesthetic): *p* < 0.024, FP(Interaction): *p* = 0.005]. A significant effect of the factor vestibular was found in one of the poster clusters [OM(Vestibular) = 0.023]. No other significant effects were detected

Subsequently, we ran *post-hoc* tests in order to examine which conditions were pairwise different. For cluster ML, *post-hoc* permutation tests showed a significant difference between passive and vestibular (*p* = 0.018), passive and active (*p* = 0.005), and vestibular and active (*p* = 0.002). For cluster FP, we found significant differences between passive and vestibular (*p* = 0.039), passive and active (*p* = 0.004), kinesthetic and active (*p* = 0.036), and vestibular and active (*p* = 0.007). In Cluster OM, we found a significant effect of passive vs. vestibular (*p* = 0.012) and kinesthetic vs. vestibular (*p* = 0.030).

Summarizing the cluster effects, we identify the following pattern: In posterior regions, the passive condition shows the weakest alpha modulation with slightly higher desynchronization in the active condition—whereas the kinesthetic and vestibular conditions display stronger modulations and therefore strong alpha desynchronization.

In more anterior regions, the fronto-parietal clusters, we see alpha synchronization in the vestibular condition, but desynchronization in the active condition. This pattern is visible in both clusters ML and MR, but only significant in ML. We conclude that differences between conditions were accompanied by significant ERSP alpha modulations in occipital and parietal regions.

## Discussion

Our study was designed with the aim to investigate the influence of different types of sensory information on EEG correlates of spatial navigation. By manipulating the availability of kinesthetic and vestibular input, we demonstrate that task-related brain activation is indeed modulated depending on the access to different sensory modalities.

We reproduced findings of earlier studies (Gramann et al., 2010; Plank et al., 2010; Chiu et al., 2012) that had shown a modulation of the alpha band in different brain areas during the turn in a triangle completion task. Furthermore, we demonstrated that incongruent information result in a modulation of alpha suppression. Depending on whether kinesthetic or vestibular information is given, medial and frontal areas show ambiguous patterns of synchronization or desynchronization. These observations reveal significant differences between the passive condition, as usually employed in laboratory setups, and the other conditions involving locomotion.

In this study, we relied on independent component analysis with subsequent source localization. Only afterwards we pooled the data (dipoles) of all subjects. This is an efficient way to deal with the small sample size. Clustering was performed by a k-means algorithm resembling the procedure in Gramann et al. (2010).

Given the experimental setup and results, there are some issues to be discussed. One might argue that the sensory impression generated by the kinesthetic condition could be artificial. Yet, it was designed in a way that the conveyed impression was as natural as possible. When using the cart and the turnable platform, the experience felt close to an on-the-spot-turn with a fixed cart, as the visual input was directly linked to the motion of the platform by an orientation sensor. We thus assume that the setup was effective in providing the desired impression of kinesthetic information in addition to vision.

Another issue and potentially confounding factor is the presence of active self-conducted and not passively initiated movement. In both the active and kinesthetic conditions, subjects had full control over their movement in the environment. In contrast to this, they had no self-control over the movement in the passive and vestibular condition. This could have been avoided by enabling the subject to navigate via joystick in the passive condition. However, in the vestibular condition active control would be more difficult to achieve. Hence, we have to bear in mind that the kinesthetic and the active condition not only include information about muscle movements, but also include cognitive processing involved in action generation as well.

Furthermore, the EEG clustering was performed not based on individual MRIs, but in a common brain space. Subsequently, we made statistical inferences on cluster level with ICs as independent measures. This implies that our statements have to be understood restricted to the specific set of subjects investigated. Future studies can improve on this situation by recruiting a representative sample of the general population and utilizing individual MRIs providing information about individual differences in subject's brain structures (Akalin Acar and Makeig, 2013).

By testing all subjects in a classical, online homing task (Goeke et al., 2013) prior to the main experiment, three of them were classified as Turners and two as Non-Turners. Subsequently, subjects exercised all four conditions of the main experiment until they felt comfortable. In the course of the actual recordings, all subjects displayed Turner behavior, i.e., the use of an egocentric reference frame, which is in line with Klatzky et al. (1998). This applied even to the two subjects that had been previously classified as Non-Turners. Their interpretation of a passive condition, akin to the typical laboratory setup, might have been influenced by performing the same task actively during the accommodation phase. Specifically, the training included active components, leading to a conflict between the movement and the mentally constructed allocentric maps. This conflict might have initiated the switch of the Non-Turner's reference frame from allo- to egocentric. Therefore, the concept of Turner and Non-Turner behavior can be interpreted as a differential involvement of distinct navigation modes in virtual environments.

We observed no distinct relationship between performance and previously classified preferred use of reference frame. It thus informs about individual preferences of spatial navigation, but not necessarily about performance in the real world (see also Klatzky et al., 1998; Goeke et al., 2013).

The overall behavioral results depict a trend toward underestimating the correct answer angle. One possible explanation could be the fact that throughout the whole experiment the arrow was initially oriented such that it was pointing away from the participant. Just as a matter of convenience or impatience, subjects might have released the button too early and thereby submitted a slightly biased, undershooting arrow adjustment. Future experiments should take this into consideration and randomize the initial orientation of the angle. An alternative explanation is that subjects simply overestimated the size of the turns.

Nevertheless, we observe a significant interaction with kinesthetic being the only condition that includes zero in the 95% bootstrapped confidence interval. As pairwise *t*-tests do not show significant differences between conditions, we can only discuss the descriptive behavioral differences. The mean relative errors of our subjects suggest a tendency toward reduced undershooting behavior in the kinesthetic condition, and is in line with the results of Frissen et al. (2011). If their findings directly transfer to our subjects, the conflicting zero-movement input from the vestibular system would lead to an underestimation of the turned angle, which should therefore elicit overshooting of the correct homing angle. An alternative explanation could be a strategy switch. In principle, reactions times could give an indication of a strategy switch. Indeed, we observe variation in reaction times with the fastest responses in the passive condition. However, in this condition subjects did not need to come to a halt. In the kinesthetic and active condition subjects had to stop by themselves and in the vestibular condition the experimenters stopped the cart. These differences can easily explain the observed variations of reaction time. Hence, interpreting the reaction time data is difficult, which leads us to refrain from a strong statement on the possibility of a strategy switch.

We did not detect consistent improvement in stochastic error in the active condition compared with the passive condition. This means that more complete navigational and congruent information did not improve the capability to choose the correct homing angle. Related results were reported by Grant and Magee (1998), who found that participants did not differ in performances when they were either actively navigating or navigating only by operating a joystick. Their performance had improved only when they carried out their task in real instead of virtual environments.

Conversely, other studies provide evidence that adding vestibular information should lead to an improved performance. Chance et al. (1998), for example, showed decreased accuracy in estimating the directions of object locations when vestibular and kinesthetic information were missing. In another study by Kearns et al. (2002), participants performed a triangle completion task either provided with visual information only, or with full bodily information from active walking. As a consequence of introducing additional kinesthetic and vestibular information, the variability of homing angle estimates decreased and general answer patterns shifted from under- to overestimation behavior. Although some participants of our study performed best in the active condition, no clear trend emerged from our data. This could be explained by a ceiling effect potentially resulting from low task difficulty.

One of our main goals was to test whether the neural correlates of spatial navigation determined in previous static EEG experiments would generalize to a more active setting. Comparing our results to past findings reveals similarities but also provides an extension of the previous literature. Nine clusters from our study closely reproduce four out of the seven clusters reported by Gramann et al. (2010). These clusters show a highly similar alpha pattern and centroid location. Similar clusters are OM, PM, MR, and PL. The cluster centroids between studies deviate to some degrees (average deviation in Talairach space: *x*: 3.75, *y*: 1.75, *z*: 9.25). This might be due to the fact that Gramann et al. visually inspected all ICs before clustering, whereas we included all computed components for clustering, and therefore added more noise to the clusters. Nonetheless, the intersection is large and clusters can be related easily. This means that the underlying sources seem to be reliable and generalize to mobile setups. The remaining five clusters are either the respective mirroring hemispheric clusters (three of five) or are new observations.

In general, the passive condition reproduces the findings by Gramann et al. (2010): Alpha activation was decreased during the turn in occipital, temporal, and parietal clusters. This suppression is thought to represent active processing and stronger cortical excitability (Pfurtscheller and da Silva, 1999; Klimesch et al., 2007) and as it was found during the turn, we conclude that the participants used more cortical resources while spatial updating was most demanding.

Even though we made some critical changes in the experimental design—we changed body position from sitting to standing, we changed the environment from a tunnel design to a starfield, we used an immersive 3D HMD instead of a computer screen, and most importantly, we used an on-the-spot-turn instead of a curved path—our results from the passive condition are nearly identical to Gramann et al.'s (2010). by means of alpha pattern and cluster locations. Due to these similarities, we argue that alpha suppression during spatial updating in a triangle completion task is a general phenomenon independent of certain changes in experimental setups.

We recorded EEG not only in the passive condition, but also in conditions where we manipulated whether kinesthetic and vestibular sensory information was provided. In posterior clusters, we found the strongest desynchronization in the vestibular and kinesthetic conditions—those that provide incongruent information about the path traveled. Conversely, in the passive condition, only moderate alpha suppression was present. In the kinesthetic and vestibular condition, the brain might need more resources to integrate partially contradictory information, like the lack of kinesthetic information or the zero-movement input from the vestibular system. The observed enhancement of alpha desynchronization could be a result of such an increased demand of resources. This pattern is present when all posterior clusters are taken into account and therefore could indicate ongoing integration processes as the parietal lobe is a prominent area for spatial navigation (Stein, 1989; Frings et al., 2006; Wolbers et al., 2007; Gramann et al., 2010) and multimodal integration (Bremmer et al., 2001). This is compatible with the observed activity in the posterior clusters, which ultimately show differential activity with different available modalities.

A different pattern emerges in anterior clusters (ML, MR, FP). The proximity to motor cortices suggests that the synchronization patterns in those clusters can be classified as mu rhythm, which is in the range of 8–12 Hz and known to get desynchronized during movement (Arroyo et al., 1993); this might account for the strong desynchronization in the active condition. In contrast to the other three conditions, the vestibular condition produces synchronization in those clusters, which might result from the absence of active movement while the participants were passively moved through space. Taken together the availability of kinesthetic and vestibular information significantly influences the pattern of alpha activity in cortical clusters.

## Concluding remarks

In this paper, we reproduced and extended previous results of Gramann et al. (2010). When only visual information was provided, we detected similar alpha band suppression during the turn of a modified triangle completion task in occipital, temporal, and parietal areas. We extended these results by providing vestibular and kinesthetic information in combination or as single, isolated sources of information. The observed difference in alpha modulations in these additional conditions demonstrates that static experiments, providing only purely visual information, omit important aspects of spatial navigation. We therefore claim that it is necessary to construct more realistic and life-like experiments to clarify the actual neural correlates behind spatial navigation. Due to rapid advances in the development of experimental equipment, this objective might become even easier to achieve in the course of the next couple of years. In regard to future studies, our approach can be applied to more complex spatial navigation tasks, like way-finding or maze tasks.

With our work, we have provided first insights into the complete picture of underlying processes and conclude that the presence of additional sensory information significantly modulates neural correlates of spatial navigation.

## Author contributions

Anna L. Gert, Benedikt V. Ehinger, Felix Weber, Gordon Pipa, Lilli Kaufhold, Petra Fischer, Peter König designed the study. Anna L. Gert, Benedikt V. Ehinger, Felix Weber, Lilli Kaufhold, Petra Fischer recorded the data. Anna L. Gert, Benedikt V. Ehinger, Lilli Kaufhold, Petra Fischer did the analysis. Anna L. Gert, Benedikt V. Ehinger, Gordon Pipa, Petra Fischer, Peter König wrote and revised the manuscript.

## Conflict of interest statement

The authors declare that the research was conducted in the absence of any commercial or financial relationships that could be construed as a potential conflict of interest.

## Supplementary material

Supplementary Figure S1 **Starfield and guiding object**. Subjects followed a small, spherical guiding object (blue) indicating the predefined path and turn. The starfield consisted of randomly distributed dots (red), which faded out within 20 m viewing distance.

Supplementary Figure S2 **Response arrow during answer period**.
