# Supplementary figures and images for: Kinesthetic and vestibular information modulate alpha activity during spatial navigation: a mobile EEG study
Source: Front Hum Neurosci. 2014 Feb 25;8:71. doi: 10.3389/fnhum.2014.00071 (PMC3934489; doi:10.3389/fnhum.2014.00071)

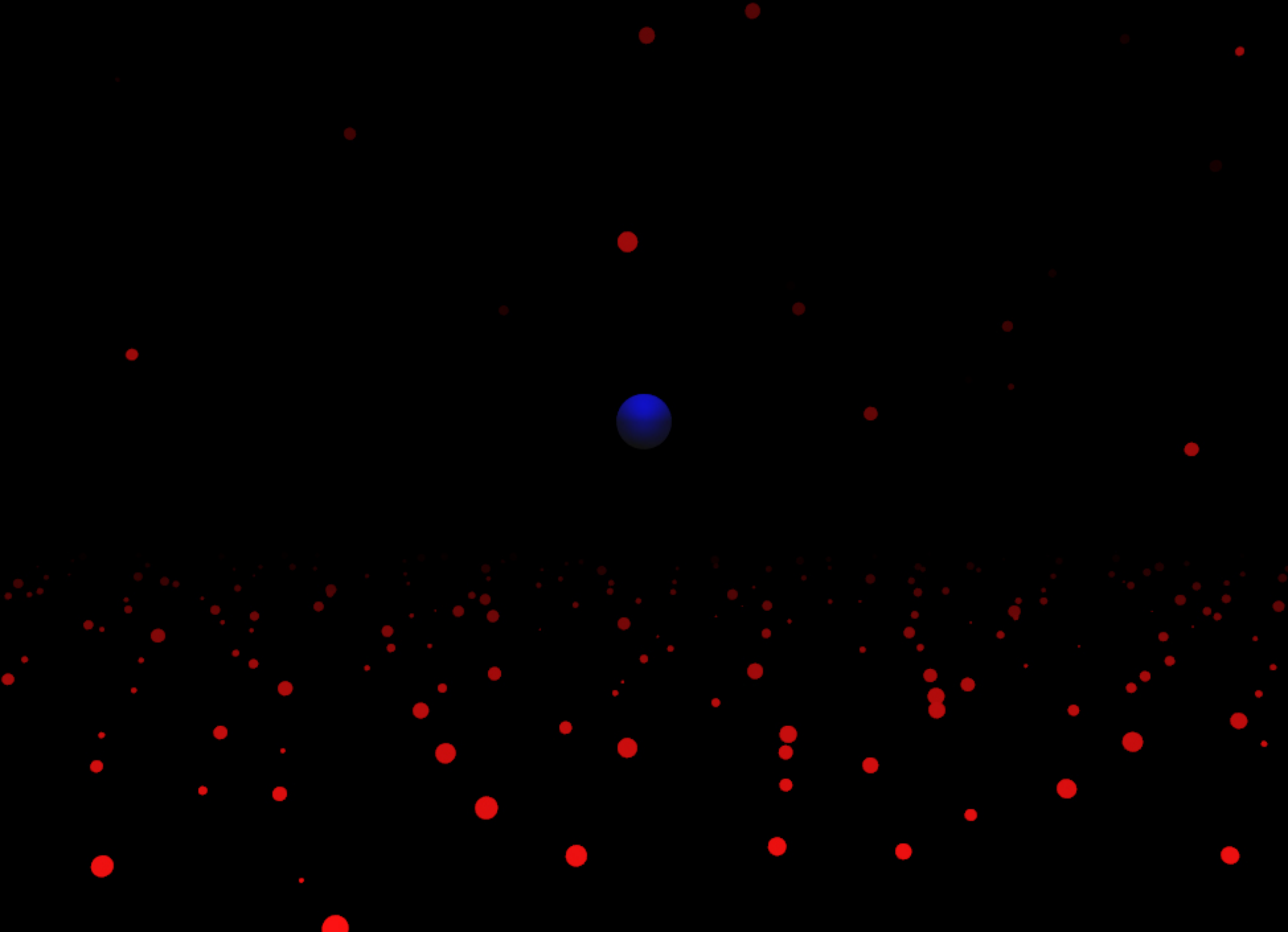

Supplement: Supplementary Figure S1 — Starfield and guiding object. Subjects followed a small, spherical guiding object (blue) indicating the predefined path and turn. The starfield consisted of randomly distributed dots (red), which faded out within 20 m viewing distance. [file Presentation1.PDF]

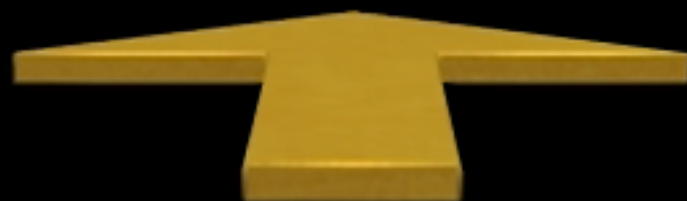

Supplement: Supplementary Figure S2 — Response arrow during answer period. [file Presentation2.PDF]
