# Supplementary material for: Kinesthetic and vestibular information modulate alpha activity during spatial navigation: a mobile EEG study
Source: Front Hum Neurosci. 2014 Feb 25;8:71. doi: 10.3389/fnhum.2014.00071 (PMC3934489; doi:10.3389/fnhum.2014.00071)
Supplement: Supplementary file 3 [file DataSheet1.DOC]

| **Name** | **Talairach Coordinates** | **Anatomical Structure** | **Broadman Area** |
| --- | --- | --- | --- |
| SC1 | -41.93, -26.69, 7.86 | Cuneus | Broadmann Area 18 |
| SC2 | -2.68, -27.32, 71.62 | Middle Temporal Gyrus | Broadmann Area 10 |
| SC3 | -1.28, 8.51, 60.56 | Middle Temporal Gyrus | Broadmann Area 39 |
| SC4 | -7.39, -6.53, 41.23 | Inferior Parietal Lobule | Broadmann Area 40 |
| SC5 | 46.39, 20.77, 30.42 | Precuneus | Broadmann Area 7 |
| SC6 | 2.59, -30.39, 17.82 | Inferior Parietal Lobule | Broadmann Area 40 |
| SC7 | -38.59, 18.77, 32.50 | Precentral Gyrus | Broadmann Area 6 |

Supplementary Table 1: X, Y, Z coordinates in Talairach space (Lancaster et al. 2000) of the seven remaining clusters, which could not be classified. (SC = Supplementary Cluster)
